# Short-term real-world outcomes of switching to faricimab in anti-VEGF-refractory retinal vein occlusion: a prospective study

**DOI:** 10.1186/s12886-026-04695-y

**Published:** 2026-02-27

**Authors:** Adnan Kilani, Abdelrahman Assaf, Denise Vogt, Efstathios Vounotrypidis, Melih Parlak, Constantin Jochem, Armin Wolf

**Affiliations:** Department of Ophthalmology, Ulm University Hospital, Prittwitzstraße 43, 89075 Ulm, Germany

**Keywords:** Anti-VEGF therapy, Angiopoietin-2/VEGF-A inhibitor, Retinal vein occlusions, Secondary macular edema, Real-world data

## Abstract

**Purpose:**

To evaluate short-term real-world outcomes after switching to faricimab using a modified treat-and-extend (TAE) regimen with a single loading dose in patients with retinal vein occlusion (RVO) refractory to prior anti-VEGF therapy.

**Methods:**

In this prospective study, 27 eyes of 27 patients with macular edema (ME) secondary to RVO and persistent intraretinal fluid (IRF) and central subfield thickness (CST) ≥ 270 μm despite ≥ 3 prior anti-VEGF injections at treatment intervals ≤ 6 weeks were switched to faricimab. Best-corrected visual acuity (BCVA) and optical coherence tomography (OCT) parameters, including CST, IRF, and subretinal fluid (SRF), were assessed from baseline (1st faricimab injection) until 3rd faricimab injection (final visit). Outcomes were analyzed for all RVO combined and stratified by central (CRVO) and branch retinal vein occlusion (BRVO).

**Results:**

In the all RVO combined cohort, median BCVA improved significantly from 0.2 logMAR to 0.1 logMAR (*p* = 0.022), and median CST decreased from 291 μm to 268 μm (*p* < 0.001) over a mean follow-up of 11.7 weeks. The proportion of eyes with IRF was significantly reduced (*p* < 0.001), and a dry macula was achieved in 48.1% of eyes at the final visit. The mean treatment interval increased significantly from 4.6 to 7.3 weeks, with an intended interval extension achieved in 88.9% of eyes. After stratification, both CRVO and BRVO subgroups showed significant CST reduction and significant treatment interval extension, while BCVA improved numerically in both subgroups without reaching statistical significance. No safety-related adverse events were observed.

**Conclusions:**

In real-world practice, switching to faricimab using a modified TAE regimen with a single loading dose appears to be effective in RVO patients refractory to prior anti-VEGF therapy, yielding significant functional improvement with early interval extension and no safety concerns.

**Clinical trial number:**

German Clinical Trials Register (DRKS) registration ID: DRKS00036984.

## Introduction

Intravitreal anti-vascular endothelial growth factor (VEGF) therapy (IVT) is considered the gold standard and first-line treatment for macular edema (ME) secondary to retinal vein occlusion (RVO) [1, 2]. However, despite advances in imaging and new established anti-VEGF regimens, a considerable number of real-world patients demonstrate suboptimal response to intensive anti-VEGF monotherapy [2, 3]. Suboptimal outcomes are primarily driven by hypoxia-induced upregulation of VEGF, which promotes vascular leakage, angiogenesis, inflammation and subsequent retinal disease progression [4, 5]. Furthermore, to achieve favorable long-term outcomes, patients are challenged with a significant treatment burden requiring frequent injections and regular ophthalmologic assessments [6, 7].

The introduction of faricimab, a bispecific monoclonal antibody that simultaneously inhibits both angiopoietin-2 (Ang-2) and VEGF-A, offers a novel dual-targeted approach for managing retinal vascular diseases and reducing treatment burden, including RVO-associated ME [8–10]. In eyes with neovascular age-related macular degeneration (nAMD) or diabetic macular edema (DME), recent real-world data confirmed that switching to faricimab in poorly responding pretreated eyes resulted in improved anatomic response and extended treatment intervals in a significant proportion of patients [11–19]. Given its recent approval for RVO-associated ME, robust clinical data on its efficacy and optimal treatment strategies in real-world setting is currently limited. In particular, optimal switch management strategies for new anti-VEGF agents have yet to be established, including omitting the loading phase during a treat-and-extend (TAE) regimen. To date, there is only one study with limited retrospectively collected real-world data demonstrating that switching to faricimab after a structured loading phase of four monthly injections resulted in significant short-term functional and morphological improvements in previously none-responsive eyes [20].

The aim of this prospective study was therefore to evaluate the short-term real-world outcomes of switching to faricimab using a TAE regimen with a single loading dose in patients with RVO refractory to prior anti-VEGF therapy.

## Materials and methods

### Participants

This prospective, real-world, monocentric study was conducted and initiated at the Department of Ophthalmology, Ulm University Hospital, Germany on January 13, 2025, and approved by the local ethics committee (application number 387/24, date of decision: January 13, 2025). Short-term outcomes of switching to intravitreal faricimab using a modified TAE regimen with a single loading dose were analyzed up to the 3rd faricimab injection (final visit) in ME secondary to RVO who were refractory to previous anti-VEGF therapy. The study was conducted in accordance with the Declaration of Helsinki and received institutional ethics approval. Prior to data collection, informed consent was obtained from the patients. The study was registered in the German Clinical Trials Register (DRKS) under the registration ID DRKS00036984.

### Inclusion and exclusion criteria

Patients with ME secondary to RVO, including central retinal vein occlusion (CRVO) and branch retinal vein occlusion (BRVO) refractory to prior anti-VEGF agents such as aflibercept 2 mg, bevacizumab, or ranibizumab were eligible. Refractive response to prior anti-VEGF treatment was defined as persistence of intraretinal fluid (IRF) with central subfield thickness (CST) ≥ 270 μm despite receiving at least three anti-VEGF injections at treatment intervals ≤ 6 weeks. Exclusion criteria included diagnosis of ocular disease other than RVO that may contribute to ME, ischemic maculopathy with foveal involvement, ellipsoid zone (EZ) disruption, iris or retinal neovascularization at baseline, baseline best-corrected visual acuity (BCVA) worse than 1.3 logMAR, significant media opacities (i.e., cataract, vitreous hemorrhage), uncontrolled glaucoma, other vitreoretinal pathology (i.e., vitreomacular traction, epiretinal membrane or macular hole on spectral-domain-optical coherence tomography [SD-OCT]), previous vitreoretinal surgery, a steroid intravitreal injection or implant at any time point.

### Treatment protocol and outcome measures

At the date of faricimab switch, prior 1st faricimab injection, a baseline visit was conducted to assess demographics (age, sex), number of previous IVTs, last anti-VEGF agent injected with last dosing interval. At baseline and subsequent visits, patients underwent a comprehensive ophthalmological assessment, including BCVA, SD-OCT, intraocular pressure (IOP) measurement, slit-lamp biomicroscopy, and funduscopy following pupil dilatation prior every faricimab injection. BCVA was measured in Snellen decimal visual acuity and converted to logMAR for statistical analysis. SD-OCT acquisition was performed with the same device from baseline (either Spectralis HRA2 + OCT, Heidelberg Engineering, or ZEISS CIRRUS 5000, Carl Zeiss AG). A comprehensive evaluation of CST changes and OCT biomarkers including IRF, subretinal fluid (SRF), hyper-reflective foci (HRF) and disorganization of the inner retinal layers (DRIL) were analyzed by OCT. After switching and receiving 1st faricimab injection, treatment interval was adjusted according to TAE regimen with an initial single loading-dose based on the presence of fluid with a CST ≥ 270 μm. Subsequent visit intervals were prolonged by two weeks in the absence of IRF or in case of reduction in retinal fluid with CST < 270 μm, and intervals were shortened by two weeks in case of deterioration with more fluid accumulation, with a minimum interval of four weeks. Outcome measures included changes in mean BCVA, mean CST, presence of OCT biomarker and intended treatment interval. The final study visit was scheduled on the day of 3rd faricimab injection, hereafter labeled as ‘visit of 3rd faricimab injection’.

### Statistical analysis

Statistical analyses were conducted using SPSS Statistics version 30.0.0.0 (IBM Corp., Armonk, NY, USA). Normality of data distribution was assessed prior to analysis, and either parametric or non-parametric tests were applied accordingly to evaluate changes in BCVA and OCT parameters from baseline to visit of 3rd faricimab injection. Depending on data distribution, continuous variables are presented either as mean ± standard deviation (SD) or as median with interquartile range (IQR). Categorical variables are reported as absolute numbers and percentages. Given the predominantly non-normal data distribution and the small size of the stratified subgroups (CRVO and BRVO), key outcome parameters including BCVA, central subfield thickness (CST), and the intended interval extension for the 4th faricimab injection were consistently reported as medians with IQRs for all groups, including the combined RVO cohort (Tables 1, 2 and 3). Comparisons between two independent groups were performed using the independent-samples t-test for normally distributed data and the Mann-Whitney U test for non-normally distributed data. Within-group changes over time were analyzed using the Wilcoxon signed-rank test. Categorical variables were compared using the chi-square test or Fisher’s exact test, as appropriate. In addition to the overall RVO analysis, outcomes were stratified by RVO subtype, separating central retinal vein occlusion (CRVO) and branch retinal vein occlusion (BRVO), as presented in Table 3. A post hoc power analysis based on an assumed effect size of 0.5, an alpha level of 0.05, and a sample size of 27 indicated an achieved statistical power of 0.81 for detecting within-group changes. As only one eye per patient was included, inter-eye correlation was not a concern in this dataset.

**Table 1:** Patient demographic and baseline characteristics at the day of 1st Faricimab injection (*n* = 27)

	All RVO combined (*n* = 27)	CRVO (*n* = 12)	BRVO (*n* = 15)	*P*-value
Mean age (SD), years	70.5 ± 11.4 (Range: 51–90)	71.6 ± 13.0 (Range: 51–90)	69.7 ± 10.2 (Range: 52–85)	0.58
Male : Female (n)	17 : 10	6 : 6	11 : 4	0.2
Phakic : Pseudophakic (n)	20 : 7	9 : 3	11 : 4	0.9
Documented history of targeted PRP (n)	10	6	4	0.2
Median number of prior anti-VEGF injections	23.0 (IQR: 15–56)	22.0 (IQR: 7–82)	23.0 (IQR: 15–44)	0.7
Last anti-VEGF agent injected prior switch (n)				
• Bevacizumab	10	5	5	0.6
• Ranibizumab	9	5	4	0.4
• Aflibercept 2 mg	8	2	6	0.2
Mean last treatment interval before switch, weeks	4.6 ± 0.8 (Range: 4–6)	4.8 ± 0.9 (Range: 4–6)	4.4 ± 0.7 (Range: 4–5)	0.2

Abbreviations: BCVA: best-corrected visual acuity; BRVO: branch retinal vein occlusion; CRVO: central retinal vein occlusion; CST: central subfield thickness;

DRIL: disorganization of the inner retinal layers; HRF: hyper-reflective foci; IRF: intraretinal fluid; IQR: interquartile range; RVO: retinal vein occlusion; SD: standard deviation;

SRF: subretinal fluid;

**Table 2:** Functional and anatomical outcomes for all RVO combined from 1st to 3rd faricimab injection visit (n = 27)

	Visit of 1^st^ Faricimab InjectionBaseline Visit All RVO combined (n = 27)	Visit of 3^rd^ Faricimab InjectionFinal Visit All RVO combined (n = 27)	P-value
BCVA, median (LogMAR)	0.2 (IQR: 0.3 - 0.1)	0.1 (IQR: 0.2 - 0.1)	0.022
BCVA, median (Decimal)	0.63 (IQR: 0.5 - 0.8)	0.8 (IQR: 0.5 - 0.8)	
CST, median (μm)	291 (IQR: 281 - 325)	268 (IQR: 253 - 289)	< 0.001
Presence of OCT biomarkers, n (%)			
• IRF	27 (100)	14 (52)	< 0.001
• SRF	1 (4)	0	1.00
• HRF	16 (59)	12 (44)	1.25
• DRIL	7 (26)	6 (22)	1.00
Mean treatment interval (SD), weeks	4.6 ± 0.8 (Range: 4 - 6)	7.3 ± 1.6 (Range: 4 - 11)	< 0.001
Median intended interval extension for 4^th^ faricimab injection (SD), weeks		2 (IQR: 2 - 4)	
Number of eyes with interval extension (%)		24 (88.9)	
Mean follow-up from the 1^st^to the 3^rd^ faricimab injection (SD), weeks		11.7 ± 2.8 (Range: 9 - 17)	

Abbreviations: BCVA: best-corrected visual acuity; CST: central subfield thickness; DRIL: disorganization of the inner retinal layers; HRF: hyper-reflective foci

IRF: intraretinal fluid; IQR: interquartile range; RVO: retinal vein occlusion; SD: standard deviation; SRF: subretinal fluid

## Results

### Demographic and baseline characteristics

A total of 27 eyes from 27 patients (17 males [63%], 10 females [37%]) were included in this prospective, real-world study (Table 1). The mean age of the study population was 70.5 ± 11.4 (range: 51–90) years. Patients had received a median of 23 prior anti-VEGF injections (IQR: 15–56) with a mean treatment interval of 4.6 ± 0.8 weeks (range: 4–6) before switching to faricimab.

Twelve eyes (44%) were diagnosed with CRVO and 15 eyes (56%) with BRVO (Table 1). Baseline demographic characteristics at the visit of the 1st faricimab injection, including the last treatment intervals before switching to faricimab, were comparable between the CRVO and BRVO subgroups, with no statistically significant differences observed (all *p* > 0.05).

The last anti-VEGF agent injected prior switching to faricimab was distributed relatively evenly: 37% (10/27) received bevacizumab, 33% (9/27) ranibizumab, and 30% (8/27) aflibercept 2 mg. History of targeted panretinal photocoagulation (PRP) was documented in 37% (10/27) of included patients (Table 1). All eyes refractory to previous IVT were switched to faricimab and treated using a modified TAE regimen with a single-injection loading phase.

### Functional outcomes

**Fig. 1:**
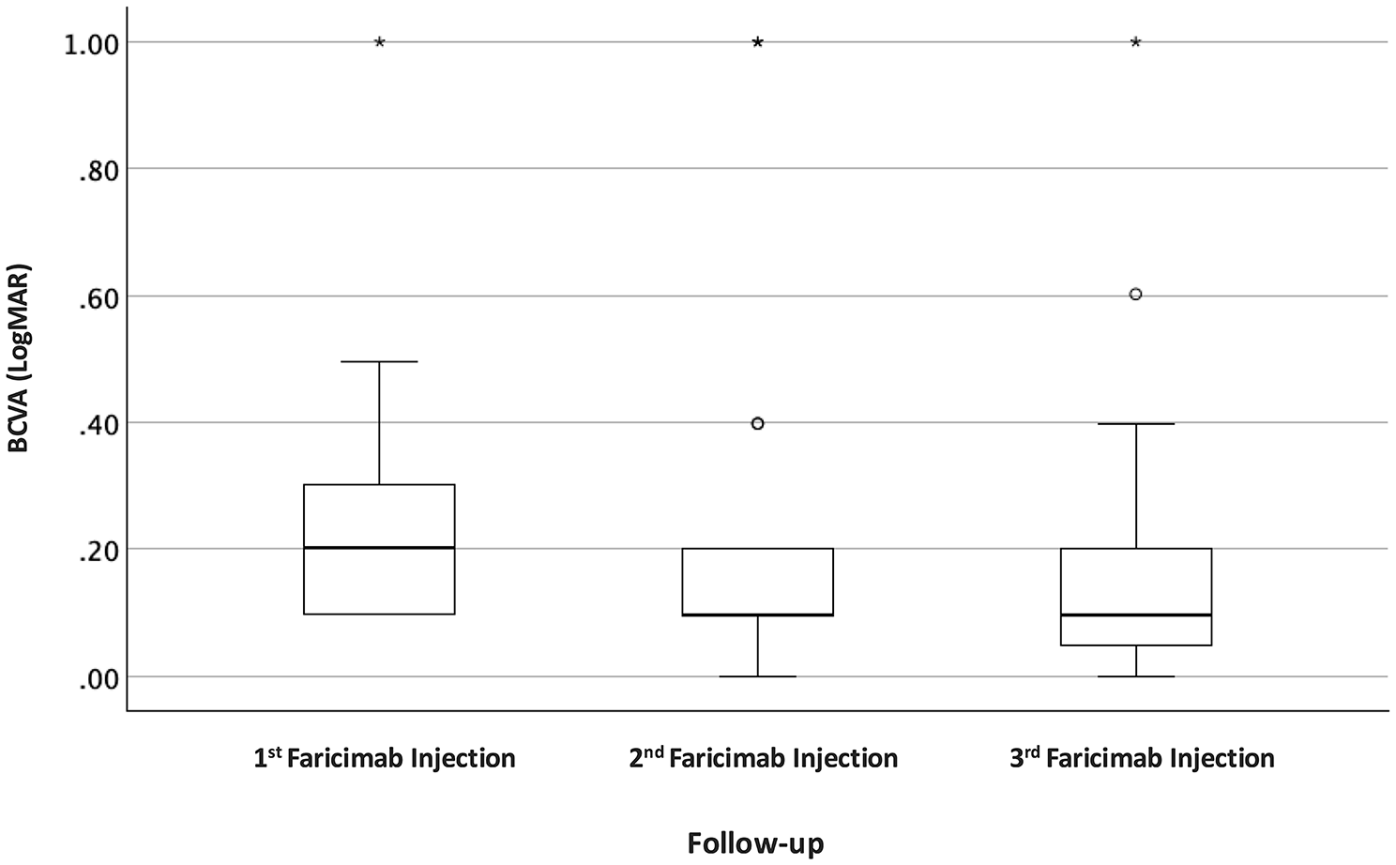
Boxplots of BCVA at 1st, 2nd and 3rd faricimab injection of all RVOs combined

In the all RVO combined cohort, BCVA improved significantly from the 1st faricimab injection visit to the 3rd injection visit. Median BCVA improved from 0.2 logMAR (IQR: 0.3 − 0.1) to 0.1 logMAR (IQR: 0.2 − 0.1) (Wilcoxon signed-rank test: *p* = 0.022) (Fig. 1; Table 2).

**Table 3:** Functional and anatomical outcomes for CRVO (n = 12) and BRVO (n = 15) from 1st to 3rd faricimab injection visit

	CRVO (n=12)			BRVO (n= 15)		
	Visit of 1st Faricimab InjectionBaseline Visit	Visit of 3rd Faricimab InjectionFinal Visit	P-value	Visit of 1st Faricimab InjectionBaseline Visit	Visit of 3rd Faricimab InjectionFinal Visit	P-value
BCVA, median (LogMAR)	0.2 (IQR: 0.3 − 0.1)	0.14 (IQR: 0.4 − 0.1)	0.1	0.2 (IQR: 0.3 − 0.1)	0.1 (IQR: 0.2–0.7)	0.1
BCVA, median (Decimal)	0.63 (IQR: 0.5–0.8)	0.8 (IQR: 0.4–0.8)		0.63 (IQR: 0.5–0.8)	0.8 (IQR: 0.6–0.8)	
CST, median (mm)	312 (IQR: 283–372)	263 (IQR: 253–276)	0.002	289 (IQR: 280–302)	270 (IQR: 253–290)	0.08
Presence of OCT biomarkers, n (%)						
• IRF	12 (100)	5 (41.7)	0.016	15 (100)	9 (60)	0.031
• SRF	0	0	--	1 (6.7)	0	1.0
• HRF	9 (75)	6 (50)	0.2	7 (46)	6 (40)	1.0
• DRIL	3 (25)	3 (25)	1.0	4 (27)	3 (20)	1.0
Mean treatment interval (SD), weeks	4.8 ± 0.9 (Range: 4–6)	7.3 ± 1.6 (Range: 4–11)	0.02	4.4 ± 0.7 (Range: 4–5)	6.7 ± 0.7 (Range: 4–10)	0.02
		CRVO (n=12)		BRVO (n=15)		
Median intended interval extension for 4th faricimab injection (SD), weeks		2 (IQR: 2–3)		3 (IQR: 1–4)		
Number of eyes with interval extension (%)		12 (100)		12 (80)		

Abbreviations: BCVA: best-corrected visual acuity; BRVO: branch retinal vein occlusion; CRVO: central retinal vein occlusion; CST: central subfield thicknessDRIL: disorganization of the inner retinal layers; HRF: hyper-reflective foci; IRF: intraretinal fluid; IQR: interquartile range; RVO: retinal vein occlusion; SD: standard deviation; SRF: subretinal fluid

Subgroup analysis demonstrated comparable functional outcomes in eyes with CRVO and BRVO. In the CRVO subgroup, median BCVA improved from 0.2 logMAR (IQR: 0.3 − 0.1) at baseline to 0.14 logMAR (IQR: 0.4 − 0.1) at the visit of 3rd faricimab injection (Table 3). In the BRVO subgroup, median BCVA improved from 0.2 logMAR (IQR: 0.3 − 0.1) to 0.1 (IQR: 0.2–0.7) at the visit of 3rd faricimab injection (Table 3). Although numerical improvements in BCVA were observed in both subgroups, these changes did not reach statistical significance when analyzed separately, likely due to the limited sample size of the stratified cohorts.

### Anatomical outcomes

**Fig. 2:**
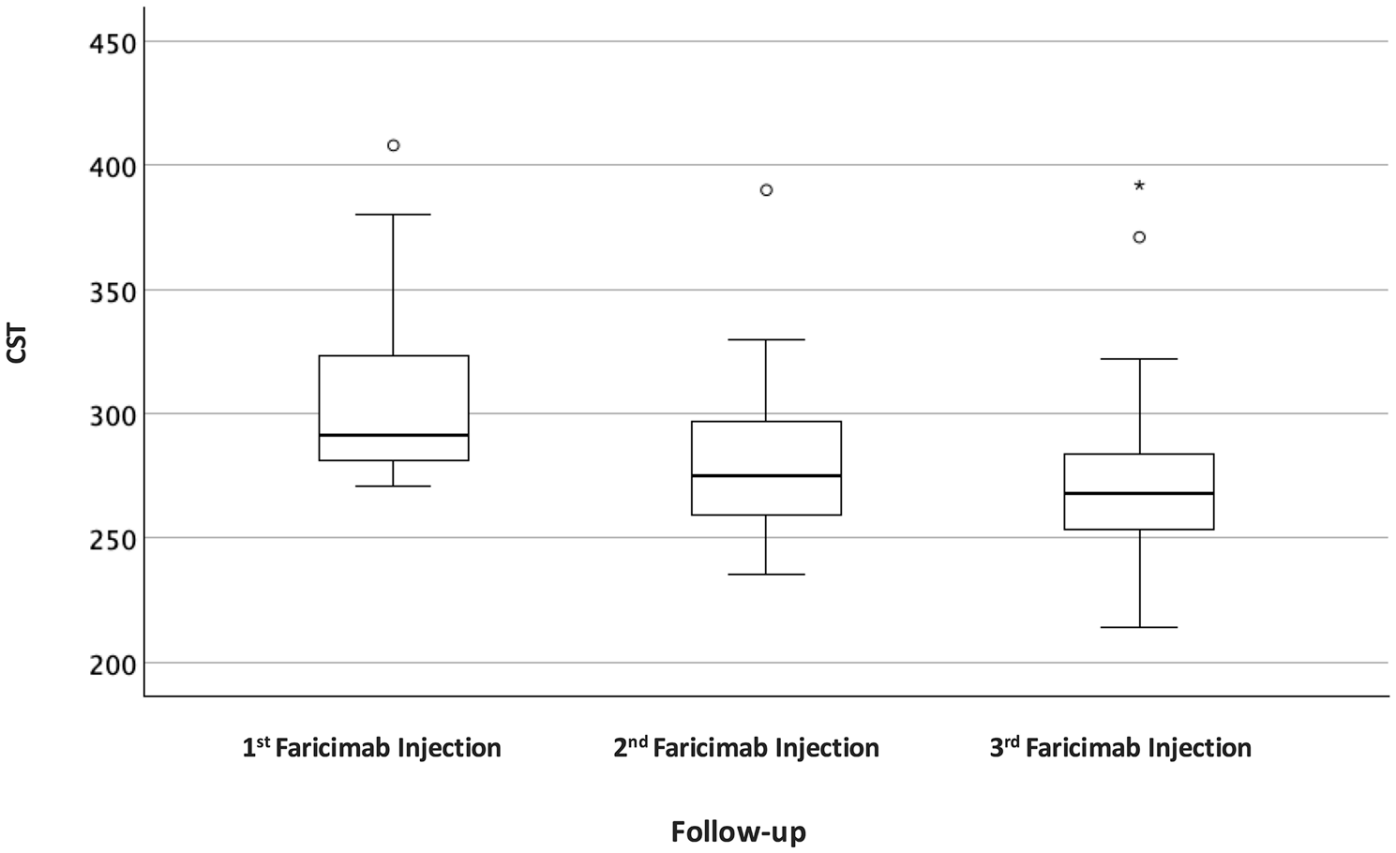
Boxplots of CST at 1st, 2nd and 3rd faricimab injection of all RVOs combined

In the all RVO combined cohort, CST decreased significantly from the 1st faricimab injection to the 3rd faricimab injection visit (Table 2). Median CST was reduced from 291 μm (IQR: 281–325) to 268 μm (IQR: 253–289) (Wilcoxon signed-rank test: *p* < 0.001) (Fig. 2; Table 2). A reduction in CST was observed in 88.9% of eyes (24/27).

At baseline, IRF was present in all eyes (100%), SRF in 1 eye (4%), HRF in 16 eyes (59%), and DRIL in 7 eyes (26%). At visit of 3rd faricimab injection, IRF persisted in 14 eyes (52%), while SRF was absent in all eyes. The reduction in IRF was statistically significant (*p* < 0.001), whereas the change in SRF was not (*p* = 1.00) (Table 2). A dry macula, defined as the absence of both IRF and SRF, was achieved in 13 of 27 eyes (48.1%).

At the same visit, HRF were observed in 12 eyes (44%) and DRIL in 6 eyes (22%). Changes in DRIL (*p* = 1.00) and HRF (*p* = 0.125) were not statistically significant. The presence of IRF at the 3rd faricimab injection visit was not significantly associated with DRIL (*p* = 0.080) or HRF (*p* = 0.547). Furthermore, CST did not differ significantly between eyes with and without IRF at this visit (*p* = 0.458).

Subgroup analysis revealed consistent anatomical improvements in both CRVO and BRVO. In the CRVO subgroup, median CST decreased significantly from 312 μm (IQR: 283–372) to 263 μm (IQR: 253–276) (*p* = 0.002). In the BRVO subgroup, median CST decreased from 289 μm (IQR: 280–302) to 270 μm (IQR: 253–290); however, this change did not reach statistical significance (*p* = 0.08) (Table 3).

In both subgroups, the proportion of eyes with IRF decreased significantly from baseline to the 3rd faricimab injection visit, while SRF was rare at baseline and absent at follow-up. Changes in HRF and DRIL within the CRVO and BRVO subgroups were not statistically significant (Table 3).

### Treatment interval extension, follow-up duration and safety

**Fig. 3:**
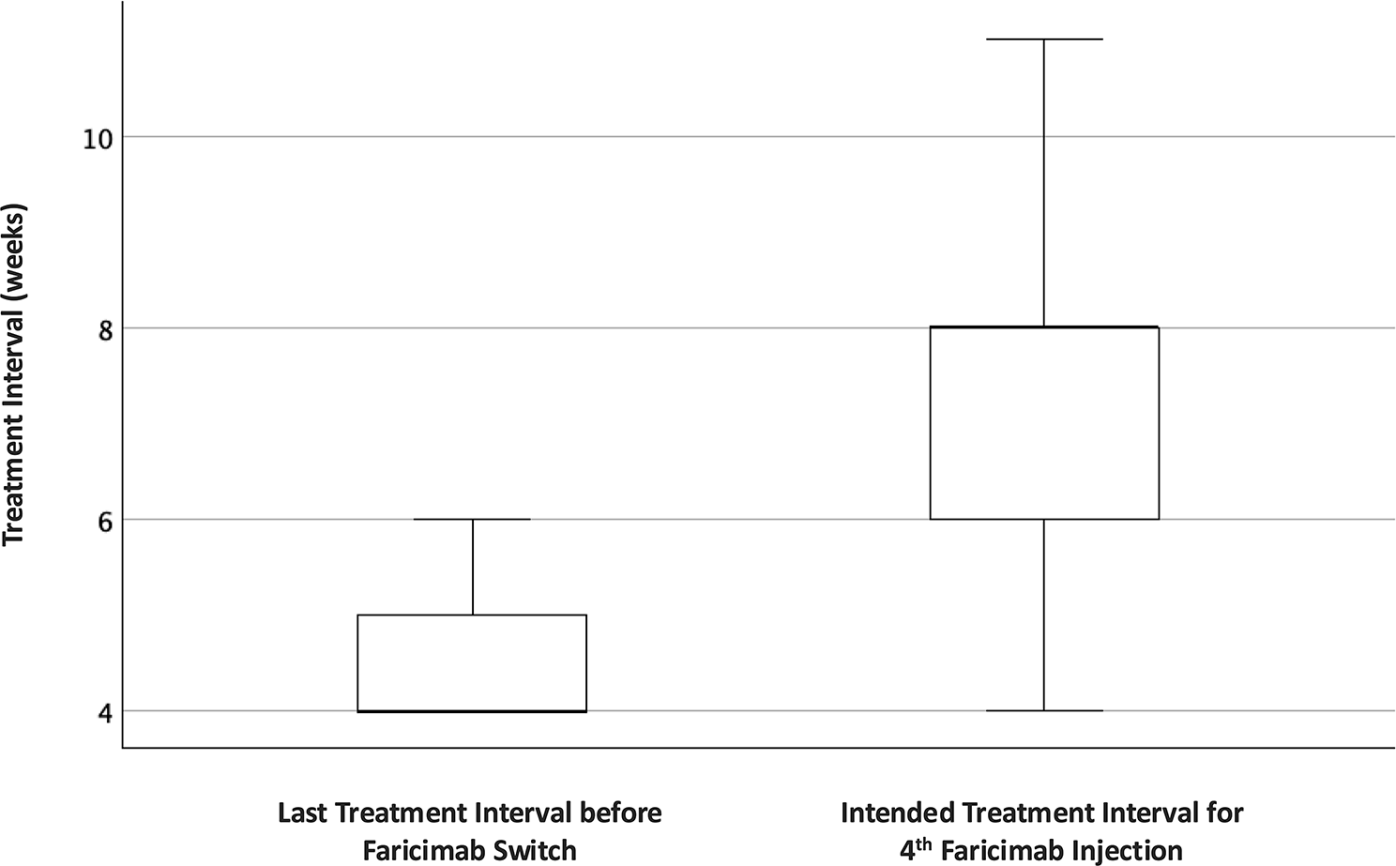
Treatment interval before and after switch of all RVOs combined

In the all RVO combined cohort, the mean treatment interval increased from 4.6 ± 0.8 weeks (range: 4–6) at the 1st faricimab injection (baseline visit) to 7.3 ± 1.6 weeks (range: 4–11) at the 3rd faricimab injection (final visit) (*p* < 0.001) (Fig. 3; Table 2). At the 3rd faricimab injection visit, the median intended treatment interval for the subsequent 4th faricimab injection was extended by 2 weeks (IQR: 2–4), and interval extension was achieved in 24 of 27 eyes (88.9%).

Stratified analysis showed comparable interval extension patterns in both RVO subtypes. In eyes with CRVO, the mean treatment interval increased from 4.8 ± 0.9 weeks to 7.3 ± 1.6 weeks (*p* = 0.02), with a median intended interval extension of 2 weeks (IQR: 2–3); interval extension was achieved in all CRVO eyes (12/12, 100%). In eyes with BRVO, the mean treatment interval increased from 4.4 ± 0.7 weeks to 6.7 ± 0.7 weeks (*p* = 0.02), with a median intended interval extension of 3 weeks (IQR: 1–4); interval extension was achieved in 12 of 15 eyes (80%) (Table 3).

The mean follow-up duration from the 1st to the 3rd faricimab injection visit was 11.7 ± 2.8 weeks (range: 9–17). During short-term follow-up, no cases of intraocular inflammation or other serious ocular adverse events related to faricimab were observed.

## Discussion

This prospective, real-world study demonstrates that intravitreal faricimab, administered using a modified TAE regimen with an initial single loading dose, results in significant short-term anatomical improvements and early extension of treatment intervals in eyes with ME secondary to RVO refractory to prior anti-VEGF therapy. At the 3rd faricimab injection visit, CST was significantly reduced (Table 2), nearly half of the eyes achieved a dry macula, and treatment intervals could be extended early in the course of intravitreal treatment (Table 2).

Importantly, these anatomical improvements and interval extensions remained consistent after stratification by RVO subtype. Both eyes with CRVO and BRVO demonstrated significant CST reduction and significant treatment interval extension (Table 3), underscoring the robustness of the observed anatomical response across clinically distinct RVO entities. In contrast, while BCVA improved numerically in both CRVO and BRVO subgroups, these functional gains did not reach statistical significance when analyzed separately (Table 3). This finding is most likely attributable to the reduced sample size following stratification and should therefore be interpreted cautiously rather than as a lack of functional treatment effect.

Managing RVO-associated ME remains challenging, particularly in patients refractory to conventional anti-VEGF agents such as bevacizumab, ranibizumab, and aflibercept 2 mg. In real-world setting, treatment burden represents a major barrier to optimal outcomes, and approximately one-third of patients with RVO-associated ME demonstrate a suboptimal response to anti-VEGF monotherapy after the loading phase or even after a year of treatment [3, 21–25]. While VEGF plays an important role in mediating the vascular permeability and neovascularization that contribute to ME, inflammation is another critical pathway contributing to the pathophysiology of ME in RVO that is not targeted by conventional anti-VEGF monotherapy [25–27]. Various studies have shown that the dual inhibition of Ang-2 and VEGF-A leads to a more durable stabilization of the retinal vessels compared to the inhibition of VEGF-A alone. Notably, Ang-2 levels are among the highest reported across retinal vascular diseases in patients with RVO [5, 28].

Faricimab, a bispecific antibody targeting both VEGF-A and Ang-2, offers a novel mechanism for stabilizing retinal vasculature by addressing both vascular leakage and inflammation [4, 5, 29]. Preclinical studies have demonstrated its ability to reverse endothelial destabilization and reduce vascular leakage, especially in ischemic retinal models [4, 5]. Clinical trials - including YOSEMITE/RHINE (for DME), TENAYA/LUCERNE (for nAMD), and BALATON/COMINO (for RVO) - have demonstrated faricimab’s non-inferior efficacy with extended durability compared to aflibercept 2 mg [8–10]. The BOULEVARD Phase II trial also showed that in pretreated DME patients, faricimab achieved longer intervals between treatments than ranibizumab [30]. Furthermore, aqueous humor analyses have demonstrated sustained suppression of VEGF-A and Ang-2 - up to 16 weeks post-dose - supporting its suitability for TAE regimens [31].

Real-world evidence on faricimab in RVO-associated ME remains limited. To date, the only available real-world study by Hafner et al. retrospectively evaluated short-term outcomes after switching to faricimab in treatment-resistant RVO following a conventional upload of three injections, without subsequent TAE implementation [20]. In contrast, our study prospectively evaluated faricimab initiation within a modified TAE strategy and demonstrated that meaningful anatomical and functional improvements with early interval extension can be achieved without a conventional loading phase, thereby potentially reducing treatment burden. Importantly, our study included a larger and more homogeneous cohort, minimizing treatment-related bias by excluding eyes with ischemic maculopathy involving the fovea or prior dexamethasone intravitreal implantation [20].

Compared with emerging real-world data in other retinal diseases, where functional gains after switching to faricimab are often modest or absent in the short term, eyes with RVO-associated ME in our cohort demonstrated pronounced anatomical improvement alongside numerically improved visual acuity [13–16, 18, 20, 32]. These findings suggest that faricimab may offer particular benefit for RVO-associated ME, likely due to the elevated Ang-2 levels characteristic of this subgroup [5, 28, 30]. By targeting both VEGF and Ang-2, faricimab may enable sustained, long-term disease control with fewer injections [18]. Furthermore, no safety concerns were observed in our study, consistent with real-world evidence across RVO and other retinal diseases, where reports of mild intraocular inflammation have been rare or absent [13, 15, 18–20, 32].

The main limitations of this study include its small sample size, which is partly attributable to the current paucity of real-world data for patients with RVO-associated ME [20]. While stratification into CRVO and BRVO subgroups reduced statistical power for functional comparisons, it represents a methodological strength of this study by allowing a differentiated assessment of treatment response across RVO subtypes with distinct natural histories.

Further limitations include, the lack of a control group, which limits comparative interpretation. Emerging agents, such as aflibercept 8 mg, have demonstrated sustained disease control in a substantial proportion of patients with nAMD (PULSAR) or DME (PHOTON) through 96 weeks with extended treatment intervals [33, 34]. While not approved for RVO-associated ME during our study period, aflibercept 8 mg may still be a promising option for RVO-associated ME. The mean follow-up period of our study group was relatively short and varied among patients, reflecting the typical challenges of real-world clinical practice. Many patients in our cohort had experienced a substantial treatment burden, with a median of 23 prior anti-VEGF injections before switching to faricimab. Inconsistent follow-up was likely influenced by reduced adherence due to chronic treatment fatigue. However, the strength in this study is our treatment protocol - initiating faricimab in a TAE regimen with an initial single loading dose - which allowed early extension of treatment intervals. Importantly, our study is among the first to prospectively evaluate short-term outcomes in a well-defined cohort of anti-VEGF refractory patients with RVO, managed using a TAE approach without the conventional loading phase. This approach supports personalized treatment approach, but contributed to the variability in follow-up.

In conclusion, our prospective real-world data indicate that faricimab, administered within a modified TAE regimen with a single-injection loading phase, provides significant short-term anatomical improvement and enables early treatment interval extension in anti-VEGF–refractory RVO-associated ME. These effects were consistent across CRVO and BRVO subgroups, while functional improvements showed favorable numerical trends without reaching statistical significance after stratification. Larger studies with longer follow-up are warranted to confirm these findings and to further clarify functional outcomes across RVO subtypes.

## Data Availability

All data supporting the findings of this study are included in the article. Due to the sensitive nature of patient data, individual-level datasets are not publicly available but may be provided in anonymized form by the corresponding author upon reasonable request.

## Abbreviations

Ang-2: Angiopoietin-2
BCVA: Best-corrected visual acuity
BRVO: Branch retinal vein occlusion
CRVO: Central retinal vein occlusion
CST: Central subfield thickness
DRIL: Disorganization of the inner retinal layers
DRKS: German Clinical Trials Register
EZ: Ellipsoid zone
HRF: Hyper-reflective foci
IRF: Intraretinal fluid
ME: Macular edema
OCT: Optical coherence tomography
PRP: Panretinal photocoagulation
RVO: Retinal vein occlusion
TAE: Treat and extend treatment
SD: Standard deviation
SD-OCT: Spectral-domain-optical coherence tomography
SRF: Subretinal fluid
IOP: Intraocular pressure
IQR: Interquartile range
IVT: Intravitreal anti-VEGF therapy
VEGF: Vascular endothelial growth factor
WHO: World Health Organization
